# Knowledge and perception of pastoral community members about brucellosis as a cause of abortion in animals and its zoonotic importance in Amibara district, Afar Region, Ethiopia

**DOI:** 10.1371/journal.pone.0206457

**Published:** 2018-11-05

**Authors:** Mengistu Legesse, Girmay Medhin, Mekonnen Bayissa, Gezahegne Mamo

**Affiliations:** 1 Aklilu Lemma Institute of Pathobiology, Addis Ababa University, Addis Ababa, Ethiopia; 2 Amibara District Pastoral Office, Afar Region, Melka were, Ethiopia; 3 College of Veterinary Medicine and Agriculture, Addis Ababa University, Bishoftu, Ethiopia; Faculty of Science, Ain Shams University (ASU), EGYPT

## Abstract

Sero-epidemiological studies of brucellosis in the Afar Region showed that the disease is prevalent in livestock. However, there is little information regarding the pastoral community members’ awareness about brucellosis as a cause of abortion in animals and its zoonotic importance. In this study, we assessed knowledge and perception of pastoral community members about brucellosis as a cause of abortion in animals and its zoonotic importance in Amibara district, Afar Region, Ethiopia. Between October and December 2016, a total of 475 study participants (age range 18–80 years, mean age 35.9 years) were interviewed about abortion in their animals, its causes, and diseases that can be transmitted to humans through consumption of raw milk. Almost all (97.7%) of the study participants reported that abortion in animals, especially in goats, is a major problem in the area, and they mentioned that disease (44.6%), drought (58.4%) and fly bites (29.5%) as the main causes of abortion. The study participantsalso thought that malaria (42.9%) and bovine tuberculosis (19.3%) can be transmitted to humans through consuming raw milk. Five respondents (4.2%) mentioned brucellosis (locally known as “hahayita”) as a disease that can be transmitted through frequent consumption of raw milk. The majority (91.9%) mentioned malaria as a cause of febrile illness in humans and 16 (4.4%) participants mentioned brucellosis as a cause of febrile illness. Some participants also mentioned brucellosis as a cause of joint swelling (hygroma) in cattle. In conclusion, the pastoral community members in the present study area lack clear understanding about brucellosis as one of the diseases that cause abortion in their animals and its zoonotic importance. There is a need to create awareness about the zoonotic and animal health importance of brucellosis through various means such as community health extension/veterinary workers and community leaders.

## Introduction

Brucellosis is an important zoonotic disease in many developing countries, which poses a significant health problem in both humans and animals. Humans acquire *Brucella* infection through consumption of raw milk, raw meat, by direct contact with infected animals’ uterine discharge, tissue or by inhaling airborne bacteria [1]. It causes a serious and debilitating disease in humans [2, 3]. Studies also indicated a high incidence of spontaneous abortion among pregnant women with brucellosis [4–6]. In humans, brucellosis is characterized by different clinical manifestations including fever, back pain, chills, headache, weight loss, muscle and joint pains, gastrointestinal complications, night sweats and loss of appetite that is difficult to distinguish clinically from other febrile illness such as typhoid fever and malaria [1, 7]. On top of this, in a country like Ethiopia where laboratory based effective diagnosis is not available, health professionals may misdiagnose brucellosis as other disease or the diagnosis may not be even considered which causes delay in providing appropriate treatment.

Brucellosis is a common disease that causes enormous economic losses in animals. It causes abortion and sterility in livestock and also reduces milk yield. The disease is highly contagious in animals because of close contact caused by the density of the flocks or herds, and the large number of organisms shed in the environment from infected animals [1,8].

In Ethiopia, sero-prevalence studies have shown the occurrence of brucellosis in animals and in patients with recurrent febrile illness in various pastoral areas of the country [9–12]. However, there is little information [13, 14] regarding the pastoralists’ awareness about the zoonotic and animal health importance of brucellosis in Ethiopia.

Pastoralists are migratory people who depend on livestock and their products, especially milk for their livelihood. The Afar pastoralists live in a semi-arid part of the country where there is the least access to health facilities in Ethiopia. They have close physical contact including handling aborted animals without protection and share water sources with their animals, both of which increase the risk for infection with zoonotic diseases like brucellosis. Hence, understanding knowledge and practices of pastoral communities about brucellosis are among the essential elements for designing effective control measures [1]. In this study, we assessed knowledge and perception of pastoral community members about brucellosis as a cause of abortion in animals and its zoonotic importance in Amibara district, Afar Region, Ethiopia.

## Methods

### Study area and population

Amibara district is found in the Middle Awash valley about 260 km to the east of Addis Ababa. The majority of the pastoral community in this district depends on livestock for their livelihoods, and recently some of the community members have started to practice growing crops like maize along river Awash. The district has a total population of 68,146 and also has a high population of livestock (cattle, goats, camels and sheep). The pastoral population of the district migrates seasonally from place to place within the district in search of water and pasture. Among other public health problems, malaria, TB, typhoid fever and water borne diseases are the leading causes of mortality and morbidity in humans in the district. The major livestock diseases in the study area are skin disease, pneumonia, pestes des petit ruminants, sheep and goat pox, foot and mouth disease [15, 16]. Detailed information on the study area and the study population has been described elsewhere [17].

### Study design, sample size estimation, and sampling procedure

Between October and December 2016, a community-based cross-sectional survey was conducted in Amibara district to assess knowledge and perception of pastoral community members about brucellosis as a cause of abortion in animals and its zoonotic importance. Sample size estimation was done based on previous information on the level of pastoral community members’ awareness about zoonotic diseases in the Afar Region [13]. Accordingly, a total of 475 participants were included in the study (assuming that 25% of the study participants have information about brucellosis, 95% confidence level, 5% degree of accuracy, a design effect of 1.5 and 10% compensation for non-respondents). The participants were eligible if they were a member of the study community, a husband/wife or other responsible person age over 18 years of age, appeared to be healthy and volunteered to participate in the study.

Sampling procedure was done according to previous study [17]. Briefly, a list of all the pastoral kebeles (the smallest administrative unit of a district) in the district was obtained from the District Health Office. Then, six pastoral kebeles were randomly selected among 14 pastoral kebeles in the district, and the selected kebeles were stratified into manageable villages. A list of households of each village was prepared. Based on the number of households in each village, the pre-estimated sample size (475) was proportionally distributed. The required number of participants (husband or wife) was selected using systematic random sampling from each village using the lists. Although it would have been ideal to include all the 14 pastoral kebeles in this study to provide general information on knowledge and perception of pastoral community members of thedistrict about brucellosis, because of financial constraints only 6 pastoral kebeles were included.

### Data collection

A questionnaire comprising both structured and open-ended questions was prepared in English (S1 File), based on information from available literature [18]. The questionnaire was translated into the national language, Amharic and pre-tested for clarity and cultural acceptability using a group of individuals consisting of health professionals, veterinarians and community members. The participants were interviewed about questions that included whether abortion is a common problem in their animals, its causes, consumption of raw milk from animals that have experienced abortion, and diseases which can be transmitted through consuming raw milk in their local language (Afar language) by trained local data collectors (who speak both Afar language and Amharic). Each interview was made by a house–to-house visit. Information on the socio-demographic characteristics of the participants was also included in the questionnaire.

During pre-testing the questionnaire, one man mentioned an illness (which is mainly characterized by joint/muscle pains and fever), locally known as hahayita. However, he or other participants did not relate the cause of hahayita with an infection, but with cold air and/or unknown cause. Interestingly, during the data collection process (almost after half of the study participants were interviewed), an old man (one of the study participants) said that this brucellosis might be a disease locally known as hahayita. He added that hahayita is a disease which can cause joint swelling in both humans and cattle, and it can be transmitted to humans through frequent consuming raw milk. Based on this information, we made some modification to the questionnaire and included questions about hahayita, its symptoms and cause.

### Ethical consideration

The study protocol was approved by the Institutional Review Board (IRB) of the Aklilu Lemma Institute of Pathobiology (ALIPB), Addis Ababa University (AAU). Permission to conduct the study was also obtained from the Amibara District Health Office. The aim of the study was explained to each of the participants and written consent was obtained from each of the study participants. Each participant was interviewed independently and the collected information was kept confidential.

### Data management and analysis

The collected data were double-entered into Epi Data software, Version 3.1, and was exported into Stata version 11 for data analysis. The proportion of respondents falling into different categories of the target questions was used to descriptively summarize the data and understand knowledge and perception of the study participants about brucellosis.

## Results

### Socio-demographic characteristics of the study participants

A total of 475 participants (age range 18–80 years, mean age 35.9 years) participated in the study. The majority of the participants were males (61.7%), married (94.3%) and illiterate (87.8%) (Table 1).

**Table 1:** Socio-demographic characteristics of the study participants.

Characteristic	Number (%)
**Gender**	
Male	293 (61.7)
Female	182 (38.3)
**Age (years)**	
18–29	161 (33.9)
30–44	203 (42.7)
45–59	80 (16.8)
60+	31 (6.5)
**Marital status**	
Married	447 (94.3)
Single	21 (4.4)
Other	6 (1.3)
**Ethnicity**	
Afar	472 (99.58)
Other	2 (0.4)
**Religion**	
Muslim	473 (99.79)
Other	1 (0.2)
**Occupation**	372 (78.7)
Pastoralist	372 (78.7)
Agro-pastoralist	94 (19.9)
Other	7 (1.5)
**Educational status**	
Illiterate	416 (87.8)
Read & write	12 (2.5)
Primary	26 (5.5)
Secondary	20 (4.2)

### Knowledge of the study participants about the cause of abortion in their animals

A majority (97.7%) of the study participants reported that abortion (locally known as “Bereate”) in animals is a major problem in the area. A majority (79.3%) of the study participants ranked abortion as the first major problem in goats, whereas, more than half (53.8%) of the participants ranked it as the second major problem in sheep (Table 2). A large proportion (88.9%) of the participants also reported that one or more of their goats aborted within the past one year. The participants mentioned disease (44.6%), drought (58.4%) and fly bites (29.5%) as the causes of abortion in their animals. They also mentioned sheep and goat pox (locally known as “Korboda”), bovine tuberculosis, malaria (locally known as “Danhaso”) and foot and mouth disease (locally called “Abibi”) as the common diseases that cause abortion. Table 2 shows participants’ knowledge about the cause of abortion in their animals.

**Table 2:** Knowledge of study participants about the cause of abortion in their animals.

Variable	Number (%)
**Abortion in animals is a common problem in this area**	
Yes	463 (97.7)
No	6 (1.3)
Not sure	5 (1.1)
**Rank abortion in goats**	
First	367 (79.3)
Second	56 (12.1)
Third	8 (1.7)
Do not know the rank	32 (6.9)
**Rank abortion in sheep**	
First	18 (3.9)
Second	249 (53.8)
Third	69 (14.9)
Fourth	13 (2.8)
Do not know the rank	114 (24.6)
**Rank abortion in cattle**	
First	61 (13.2)
Second	69 (14.9)
Third	190 (41.0)
Fourth	34 (7.3)
Do not know the rank	109 (23.5)
**Rank abortion in camels**	
First	15 (3.2)
Second	15 (3.2)
Third	21 (4.5)
Fourth	165 (35.6)
Do not know the rank	247 (53.3)
**The number of your animals aborting within the past one year**	
One or more goats	401 (88.9)
One or more sheep	280 (78.4)
One or more cattle	256 (56.9)
One or more camels	87 (42.0)
**Cause of abortion in your animals**	
Diseases	186 (44.6)
Drought	237 (58.4)
Fly bites	119 (29.5)
Other factors like strong sun light, migration	82 (19.7)
Do not know	70 (16.8)
**If the cause was a disease, name of the disease**	
Sheep and goat pox (Korboda)	59 (31.2)
Bovine tuberculosis	34 (18.0)
Malaria (Danhaso)	22 (11.6)
Foot and mouth disease (Abibi)	11 (5.8)
Do not know its name	87 (46.0)

### Participants’ knowledge and perception about diseases which can be transmitted to humans through consuming raw milk

Table 3 shows participants’ knowledge and perception about diseases that can be transmitted to humans through consuming raw milk. A majority (91.9%) of the study participants reported that they consume raw milk from cows, goats, camels and sheep. However, almost all (99.8%) of the study participants reported that they do not consume raw meat. A considerable number of the study participants, 120 (26.1%) thought that some diseases can be transmitted through consuming raw milk. Malaria (42.9%) and bovine tuberculosis (19.3%) were mentioned as diseases that can be transmitted through consuming raw milk from sick animals, while only 5 (4.2%) individuals mentioned brucellosis (locally known as “Hahayita”). A majority (90.1%) of the study participants had no information whether consuming raw milk from animals that have experienced abortion can transmit a disease or not. Moreover, a majority (90.9%) of the study participants mentioned that they had contact with an aborted fetus or uterine discharge and retained placenta from an aborted fetus or animals that give birth and wash their hands with water (81.7%) if available; otherwise they rub their hands using soil.

**Table 3:** Knowledge and perception of study participants about diseases transmit to humans through consuming raw milk.

Variable	Number (%)
**There is a disease that can be transmitted from animals to humans through consuming raw milk**	
Yes	120 (26.1)
No	230 (50.0)
Do not know	110 (23.9)
**If yes, mention the disease**	
Malaria	51 (42.9)
Bovine tuberculosis	23 (19.3)
Brucellosis (Hahayita)	5 (4.2)
Others like sheep and goat pox	11 (9.2)
**Do you/your family drink raw milk from animals experienced abortion?**	
Yes	444 (97.6)
No	11 (2.4)
**Do you think that a disease which causes abortion can be transmitted to humans through consuming raw milk?**	
Yes	45 (9.9)
No	410 (90.1)
**If yes, name of the disease(s)**	
Brucellosis (Hahayita)	13 (28.9)
Malaria, tuberculosis, sheep and goat pox	**7** (15.6)
Do not remember the name of the disease	26 (57.8)
**Have you ever touched uterine discharge or aborted animals?**	
Yes	420 (90.9)
No	32 (6.9)
Do not remember	10 (2.2)
**Do you think that some diseases can be transmitted to humans through touching uterine discharge or aborted animals?**	
Yes	63 (13.6)
No	149 (32.3)
Do not know	250 (54.1)

### Knowledge of the study participants about diseases which cause febrile illness in humans

Table 4 depicts the study participants’ knowledge about some diseases which cause symptoms like fever, joint pain, headache and back pain in humans. A majority (80.4%) of the participants reported that they knew some diseases which cause illness like fever, joint pain, headache and back pain, while most of them (91.9%) mentioned malaria and cold air (28.1%). Only 16 (4.4%) participants mentioned brucellosis (locally known as Hahayita) as a cause of febrile illness. However, only 4 (0.9%) individuals reported that they heard about a disease called brucellosis. On the other hand, among 206 participants who were asked whether they have ever heard about a disease locally known as hahayita, 184 (89.3%) reported that they heard it. Out of these 184, 178 (96.7%) also responded that they knew the symptoms of hahayita. The study participants mentioned joint pain and knee swelling (97.8%) as the common symptoms of hahayita in humans. Some of the participants (10.1%) also mentioned that this disease (hahayita) can affect animals and it causes joint swelling (hygroma) especially in cattle. However, only 24 (13.5%) individuals mentioned that frequent consuming raw milk can cause hahayita.

**Table 4:** Knowledge of the study participants about diseases which cause febrile illness in humans in the study area.

Variable	Number (%)
**Do you know some diseases which cause illness like fever, joint pain/swelling, headache and back pain in humans?**	
Yes	360 (80.4)
No	88 (19.6)
**If yes, mention some of them**	
Malaria	331 (91.9)
Cold air	101 (28.1)
Typhoid	54 (15.0)
Hahayita (Brucellosis)	16 (4.4)
Others such as tuberculosis, amoeba and common cold	51 (14.2)
**Have you ever heard a disease called brucellosis?**	
Yes	4 (0.9)
No	462 (99.1)
**Have you ever heard a disease locally known as hahayita?**	
Yes	184 (89.3)
No	22 (10.7)
**Do you know symptoms of hahayita (n = 184)?**	
Yes	178 (96.7)
No	6 (3.3)
**Symptoms of hahayita:**	
Joint pain and knee swelling in humans	174 (97.8)
Joint swelling in cattle	18 (10.1)
Other symptoms like headache, fever, weakness, back pain	17 (9.6)
**Cause of hahayita**:	
Cold air	50 (28.1)
Frequently consuming raw milk	24 (13.5)
Other factor such as blood contamination, accident and work load	21 (11.8)
Do not know	93 (52.3)

## Discussion

In this descriptive pastoral community-based cross-sectional study, an attempt was made to assess whether abortion is a major problem in their livestock, community members’ knowledge about brucellosis as a cause of abortion in animals and its zoonotic importance, mode of transmission from animals to humans and risk practices for acquiring *Brucella* infection in humans. Almost all the study participants reported that abortion in livestock, mainly, in goats and sheep is a major problem in the study area. Most of them also reported having one or more aborted goats, sheep, cattle and camels within the past last year (Table 2). Some of the participants mentioned diseases like sheep and goat pox, bovine tuberculosis, malaria and foot and mouth disease as the common diseases that can cause abortion in their animals. Although the main diseases which cause abortion in animals in the present study area need further investigation, previous sero-epidemiological studies of brucellosis in the Afar Region indicated that the disease is widespread among small ruminants [11, 19], especially in animals with history of abortion [20]. Several studies have also isolated *Brucella* species mostly *B melitensis* from biological samples like tissue, milk, vaginal swabs, uterine fluid and/or blood samples collected from goats and sheep with history of recent abortion in different endemic countries suggesting that brucellosis is one of the diseases which causes abortion in small ruminants [21–24].

A considerable number of the study participants argued thatsheep/goat pox which causes skin lesions and affects both sheep and goats is among the diseases that cause abortion in their goats and sheep. Studies also showed that sheep or goat pox is endemic in Northern and Central Africa, Middle East and Asian countries where it causes a significant abortion and mortalityin small ruminants [25, 26]. Some of the study participants also raised the possibility that foot and mouth disease is another disease which causes abortion in their cattle. The pastoral community members’ concern about foot and mouth disease as a cause of abortion in their cattle is similar to the findings of a study by Kothalawalaet al. [27] in Sri Lanka where farmers identified this disease as a cause of abortion in their cattle, but they had no adequate information regarding brucellosis as a cause of abortion in animals. Evidence also shows that foot and mouth disease causes abortion in cattle [28, 29].

The community members in the present study area also thought that malaria can affect their cattle; it causes abortion and can be transmitted to humans through consumption of raw milk. The community members know malaria as an illness characterized by symptoms like fever, joint pain, headache and back pain. There are also several zoonotic diseases like Q fever, campylobacteriosis and leptospirosis which cause abortion in animals and can be transmitted to humans through consumption of raw milk and manifest symptoms similar to that of malaria in humans [30]. The non-specific symptoms of malaria could lead to the misconception reflected in this study as the pastoral community members might not have information on other zoonoticdiseases which cause similar illness with malaria and this issue warrants further studies. Similar to the concern of the study participants in the present study area, in Tanzania, community members suggested malaria as the most common cause of fever in humans, while a hospital based evidence indicated that bacterial zoonoses were the most common cause of fever among patients [31].

On the other hand, none of the respondents mentioned brucellosis (hahayita) as a disease which causes abortion in animals in the present study, and the finding of our study contrasts the findings of studies from elsewhere [32,33] which showed that most of the study participants mentioned abortion as the major clinical sign of brucellosis in animals. In the present study, pastoralists’ lack of awareness about brucellosis as one of the diseases which cause abortion in their livestock could imply lack of a well organized extension system (through community veterinarian workers and media) to create awareness to the community members about diseases which cause abortion in their animals like elsewhere [33]. The Afar pastoralists have a traditional way of exchanging information (locally known as *Dagu*) through the relaying of news about important events such as social, political or diseases from one person to another [34]. Maintaining such culture, promoting its use to disseminate important health issues in both humans and their livestock by community health workers could have a significant role in creating awareness among the pastoralists.

Brucellosis is are-emerging zoonotic disease that causes a considerable human morbidity in many areas of the world [2, 3]. However, the results of this study indicated that the pastoral community members in the present study area had no information on the zoonotic importance of brucellosis as very low percentage (4.4%) of the participants mentioned hahayita (brucellosis) as febrile causing illness in humans. Moreover, the study indicated that the word brucellosis is not familiar to the pastoral community members in the present study areas, as a majority (99.1%) of the participants reported that they had never heard of a disease called brucellosis. This is similar to the finding of a previous study done in the Afar Region in which only 13 (7.7%) study participants reported that they had ever heard about a disease called brucellosis [14]. The result is also in agreement with the findings of a study from Tajikistan in which the majority (85%) of the participants had never heard of brucellosis [35]. A study by Kothalawala et al. [27] in Sri Lanka also demonstrated that only 2.6% of the study participants knew the zoonotic importance of brucellosis.

In contrast to this finding, studies done in other countries such as Kenya [32], Uganda [18] and Jordan [36] indicated that a large proportion of the study participants knew the disease by the name brucellosis. In the present study area, there was no laboratory based facility for the diagnosis of brucellosis in humans and even health professionals are not clearly aware of the problem and this could be one possible explanation as to why most of the pastoral community members are not aware of brucellosis in the present study area. Moreover, the non-specific clinical signs of brucellosis in humans are major challenge for health professionals in properly diagnosing and treating patients with symptoms such as fever, headache, joint pain and back pain which are often misdiagnosed as malaria or typhoid fever [37].

Although the study participants had not heard of a disease known as brucellosis like TB or typhoid, a majority (89.3%) of the study participants (among those individuals who were asked about hahayita) reported that they know a disease locally known as hahayita and also its main symptoms (joint pain and knee swelling). Compare to the knowledge of the Amibara pastoralists regarding the symptoms of hahayita (brucellosis) in a study by Kansiime et al. [18] in Uganda, a majority of the study participants (75.5%) reported joint and muscle pain as common symptoms of brucellosis in humans.

On the other hand, similar to previous reports from Ethiopia [13,14], Sri Lanka [27] and Nigeria [33]most of the study participants had no a clear information regarding the zoonotic importance and mode of transmission of the disease (as only 24 individuals mentioned that frequently consuming raw milk could cause hahayita), while they practice risk factors like touching aborted fetus, uterine discharge or retained placenta or aborted animals without protection and also consume raw milk from animals which have experienced abortion. This finding contrasts with the findings of studies from Uganda [18], Jordan [36] and Kenya [32] in which the majority of the respondents suggested consumption of unpasteurized dairy products as the main routes of infection in humans. This lack of information about the zoonotic importance of hahayita (brucellosis) and its mode of transmission could hamper the prevention and control of the disease in the present study area.

### Limitations

This study would provide information on the level of knowledge and perception of the studied community members about the zoonotic and animal health importance of brucellosis where such data was not available. However, the survey was conducted only in one district of the Afar Region which was conveniently selected, and this would affect the generalization of the findings to other pastoral communities of the Afar Region. In addition, this quantitative study was not supplemented by qualitative study like focus group discussion which is important to gather detailed and additional information regarding community’s knowledge about brucellosis and other diseases which cause abortion in animals and also affect humans.

### Conclusion

The study participants in the present study area reported that abortion is a major problem in their livestock mainly in goats and sheep. However, the findings of the present study showed that the pastoral community members had no adequate information regarding brucellosis (hahayita) as one of the diseases which cause abortion in their animals. Although the community members consider hahayita (brucellosis) as a disease that causes joint and knee swelling in both humans and animals, most of them had no clear information on its zoonotic importance and mode of transmission. Thus, there is a need to increase knowledge of community members about the zoonotic and animal health importance of brucellosis using various strategies like disseminating information through community health extension/veterinary workers, community leaders and “Dagu” system. Advising the importance of boiling milk as a preventive method for many diseases which can be transmitted to humans through consuming raw milk would have also great importance.

## Supporting information

S1 File Questionnaire used in the study to assess the awareness of pastoral community about brucellosis in Amibara district, Afar Region, Ethiopia. (DOCX)
