# Supplementary material for: Knowledge and perception of pastoral community members about brucellosis as a cause of abortion in animals and its zoonotic importance in Amibara district, Afar Region, Ethiopia
Source: PLoS One. 2018 Nov 5;13(11):e0206457. doi: 10.1371/journal.pone.0206457 (PMC6218040; doi:10.1371/journal.pone.0206457)
Supplement: S1 File — (DOCX) [file pone.0206457.s001.docx]

Questionnaire for assessment of pastoral community’s awareness about brucellosis in Amibara District, Afar Region

Exclusion criteria = urban residents, individuals cannot hear, speak, sick individuals, children age less than 18 years, non-residents of the selected study area,

**Part I. Sociodemographic characteristics of the respondents**

1.Name of respondent: ____________________ Kebele ____________________, village _____________________________________

1. Sex **:** 1= Male 2= Female
2. Age (Year): ____________

4. Ethnicity**:** 1= Afar 2= other_________________

5. Religion 1= Muslim 2= Protestant Christian 3=Orthodox Christian 4= other specify _____________

6. Marital status 1= Married 2= single 3 = divorced 4= widowed

7. Educational status: 1= illiterate 2= can read only 3 = read and write 4= secondary (9- 12) 5= other (specify) ______________________________

8.Occupation: 1= nomadic-pastoralist 2= agro-pastoralist 3= daily laborer 4=

5= Jobless 6= other (specify) ____________________

Part II**. Questionnaire to assess the knowledge of the respondents about brucellosis**

1. **Is abortion/still birth (locally known as “Bereate) in goats , sheep, cattle and camels a common problem in this area?**

1. Yes 2 . No 3. Do not know

2**. If yes, in which animals it is most common in this area (rank it as first , second , third , fourth and I do not know the rank)?**

**2.1. Rank abortion in goats** : 1. first 2. second 3. third 4. fourth 5’.I do not know the rank

**2.2. Rank abortion in sheep**: 1. first 2. second 3. third 4. fourth 5. I do not know the rank

**2.3. Rank abortion in cattle:** 1. first 2. second 3. third 4. fourth 5. I do not know the rank

2.4. **Rank abortion in camels**: 1. first 2. second 3. third 4. fourth 5. I do not know the rank

3. **How many of your goats, sheep, cattle and camels have experienced abortion / still birth/ in the last one year?**

1. Goats _______ 2. Sheep __________ 3. Cattle __________4. Camels__________

4. **What do you think the cause of the abortion in your animals ?**

1. disease (s) 2. accident 3. shortage of food (drought) 4. Flies/insects bite 5. Other factors , specify _______________________________________________

6. Don know the cause of abortion

1. **If a disease, what is the name of the disease(s)__________________________?**

6. **Do** **you/your family drink raw milk from cattle, goat , sheep and camel** ?

1. Yes 2. No

7. **Do you/your family consume raw meat** ? 1. Yes 2.No

**8. There is a disease that can be transmitted from animals to humans through drinking a raw milk**: 1. Yes 2. No 3. Do not know

9. **If yes , mention the name of that disease**(s)_____________________________

10. **Do you/your family drink raw milk from animals experienced abortion**?

1.Yes 2.No

11. **Do you think that a disease which causes abortion can be transmitted to humans through consuming raw milk ?** 1.Yes 2. No

12. **If yes, name of the disease(s)** ___________________________________

13. **Do you touch aborted fetus/ retained placenta/uterine discharge with your hand without proper protection?**

1. Yes 2. No 3. Do not remember

14. **Do you think that some diseases can be transmitted to humans by touching uterine discharge or aborted animals?** 1. Yes 2. No 3. Do not know

**15. Do you wash your hand after touching aborted animals or uterine discharge?**

1. Yes alway 2. No

16. **Do you know some diseases which cause illness like fever, joint pain/swelling, headache and back pain in humans ?** 1. Yes 2. No

17. **If yes, mention some of them:_______________________________**

18. **Have you ever heard a disease called brucellosis?** 1. Yes 2.No

19**. If yes, from whom have you heard about it ?**

1. friend (s) 2. health workers 3. media (radio, TV poster etc)

4. veterinarians 5. traditional healers

6. Other (specify)_______________________

20. **If yes to question 18, do you know the symptoms of brucellosis in humans ?**

1. Yes 2= No

21. **If yes, what is/are the symptoms of brucellosis in humans ?**

1. Back pain 2. Fever, 3. Vomiting 4. Headache 5. Malaise

6. Other specify _____________________________________________

22**. What is the cause of brucellosis**? ____________________________

23**. Have you ever heard about a disease locally called hahayita** ?

1. Yes 2. No

24**. If yes, do you know symptoms of hahayita in humans** ? 1. Yes 2. No

25. **If yes, symptoms of hahayita** ? ____________________________________

26. **What is the cause of hahyita** **in humans**?__________________________________

27. **If you have any additional idea or opinion?** _____________________________

Thank you !
